# Implementation and Integration of Regional Health Care Data Networks in the Hellenic National Health Service

**DOI:** 10.2196/jmir.4.3.e20

**Published:** 2002-12-31

**Authors:** Petros Lampsas, Ioannis Vidalis, Christos Papanikolaou, Aristides Vagelatos

**Affiliations:** ^1^University of ThessalyComputer and Communications Engineering DepartmentVolosGreece; ^2^Research Academic Computer Technology InstituteDepartment of IT Projects for the Public SectorAthensGreece

**Keywords:** Computer communication networks, network architecture and design, computer security, internetworking, medical informatics, information systems, health information systems

## Abstract

**Background:**

Modern health care is provided with close cooperation among many different institutions and professionals, using their specialized expertise in a common effort to deliver best-quality and, at the same time, cost-effective services. Within this context of the growing need for information exchange, the demand for realization of data networks interconnecting various health care institutions at a regional level, as well as a national level, has become a practical necessity.

**Objectives:**

To present the technical solution that is under consideration for implementing and interconnecting regional health care data networks in the Hellenic National Health System.

**Methods:**

The most critical requirements for deploying such a regional health care data network were identified as: fast implementation, security, quality of service, availability, performance, and technical support.

**Results:**

The solution proposed is the use of proper virtual private network technologies for implementing functionally-interconnected regional health care data networks.

**Conclusions:**

The regional health care data network is considered to be a critical infrastructure for further development and penetration of information and communication technologies in the Hellenic National Health System. Therefore, a technical approach was planned, in order to have a fast cost-effective implementation, conforming to certain specifications.

## Introduction

It is widely recognized that utilization of information and communication technologies (ICT) can be of great benefit for the Hellenic, as well as any other, National Health System (NHS). The current penetration of information and communication technologies in the Hellenic health care sector can be summarized as:

- lack of appropriate infrastructure, applications, and specialized personnel
- fragmentary use of information and communication technologies in key sections, where the advantages would be straightforward.

Projects such as installing health information systems, developing dedicated health portals, and implementing related training programs are expected to reduce expenses and significantly improve clinical and administrative services.

Providing interconnection capabilities to health care establishments will serve not only the Hellenic National Health System administration mechanism, but also medical care itself. The term *interconnection capabilities* includes the Internet connections of hospitals and administration units, the participation of general health care service-provision units in health care networks, the installation and operation of regional telematic applications, and the development of health-information sources for consumers.

For the development of health-information sources for consumers, validity and non-repudiation (that is, authentication that with high assurance can be asserted to be genuine, and that cannot subsequently be refuted) of health-related information should be ensured. The other capabilities presume the existence of a *secure private* network maintained by a management and technical-support mechanism to make their use effective.

By *secure* we mean that the network should reinforce the protection of sensitive information. The characterization *private*(which does not necessarily mean privately owned) encompasses quality of service, availability, and technical support.

In this paper, we present the proposed design for implementing a secure, cost-effective nationwide data-network infrastructure for the functional interconnection of health care and administration units. This infrastructure will facilitate the exchange of medical information, the exchange of administrative information, and the support of regional telematic applications (eg, telemedicine services in difficult-to-approach areas), wherever required.

## Methods

Since 2001, a reformation aimed at the overall improvement of the Hellenic health care sector has been evolving. One of the main changes, directly affecting the framework examined, is the division of the country into 17 autonomous health care regions. Each region has its own administrative structure, supervises all the health care units that reside in its territory, and typically includes 6 to 10 state hospitals, 10 to 15 primary health care units, and 15 to 20 community clinics. The head department (regional health authority) is responsible for the delivery of high-quality health services to the people.

The main objectives related to implementing a secure network infrastructure for health authorities are on the one hand tied up with certain factors of the above-mentioned reformation of the Hellenic health care sector and on the other hand tied up with major advances that make use of information and communication technologies essential in such a field (eg, development of medical portals and the worldwide trend for patient-centered care) [1]. Table 1 lists the factors that lead to the need to implement this health-authorities' interconnection infrastructure. These factors may also be seen as the targets that will eventually be accomplished by the Hellenic National Health System.

**Table 1:** Benefits of using information and communication technologies in the health care sector

**Health Care Players**	**Benefits**
Administration units	Policy development and decision-making are strongly supported by effective and on-time information gathering and distribution. Easier adaptation to eEurope challenges. Supply control; better budget monitoring. Overall improvement in the way citizens are served.
Hospitals	Increased efficiency in communication between hospitals, administration units, social security services, careers, physicians, and citizens. Personnel familiarization with information technologies through Internet-access operations. Patient-record traffic support. Reinforcement of the need to build health care information systems (HCISs) and local networks in hospitals. Utilization of the developed Intranets. Better information services for the citizens. Advanced telematic services (eg, telemedicine applications in difficult-to-reach regions).
Health care personnel	Meets the increased need for telecommunications not only for medical, but also for compensation reasons. Participation in care chains and relevant coordination. Physicians' collaboration. Patients'-history data retrieval. Continuing education services; familiarization with new technologies through special training programs. Interaction with patients to provide advice or prescriptions.
Citizens	Use of the Internet for health-related information retrieval. Information and communication technologies will increase interest in citizens' health-issues management. Creation of the appropriate infrastructure for future provision of special health services for specific population groups (eg, in-house services for older people or patients with long-lasting attendance and nursing needs).

Table 1 constitutes the functional requirements for the network infrastructure and includes elements of the above-mentioned evolving reformation and of current advances in the field of data communications. Therefore, certain factors, such as:

- the regional structure of the administration services
- the treelike managerial structure of the health care units
- the active participation of general practitioners in the new system
- the need for transmission of sensitive electronic health record (EHR) information and for interconnection with private pharmacies and the insurance system in the years to come
- the successive advances in Internet technologies

lead us to consider secure transmission over public networks - eg, over the Internet using VPN (Virtual Private Network) technologies - as the best implementation solution.

By establishing end-to-end secure links among multiple sites in a public network, VPNs are the answer to the very-expensive solution of using dedicated leased lines for enterprises' and organizations' data communications [2]. Recently, the shared infrastructure across which this alternative way to build a private network operates tends to be the Internet. The Internet is an obvious choice for the realization of a VPN, because most organizations' intranets and collaborating establishments' extranets use TCP/IP (Transmission Control Protocol over Internet Protocol) technologies to exchange data and almost every organization possesses at least one Internet connection. Other factors contributing to the selection of the Internet are the recent advances in data networking, such as the ability to transmit real-time traffic using the TCP/IP protocol suite.

The evaluation of the success or lack of success of such a solution is going to be based on the following major technological and functional criteria for the implementation of the health authorities' network infrastructure [3]:

- security, one of the most important requirements when dealing with extremely-sensitive data, like health care records
- quality of service (QoS), especially important in certain applications (eg, teleconsultation or telesurgery)
- implementation period, which should be the shortest possible, since the Hellenic National Health System has to stay competitive with the private sector
- technical support schema, a matter essential in our case, because of the lack of information technology personnel in Hellenic health care units [4]
- backbone and distribution-network bandwidth (capacity for data transfer), which should be the highest possible, while keeping implementation and operating costs low
- ability to provide regional telematic services, to support - among other things - the new regional structure of the Hellenic National Health System
- ability to incorporate technology improvements.

Regional Health Care Data Network Infrastructure: Main Characteristics

As mentioned earlier, the proposed solution comprises use of the Internet and realization of VPN technologies.

The regional health care data networks will encompass the following technologies related to common VPN architectures [5- 7]:

- remote access VPNs, which extend the corporate network to telecommuters, mobile workers, and remote offices, enabling users to connect to their enterprises' intranets and extranets, using access methods such as dial-up, ISDN (Integrated Services Digital Network), and wireless mobile IP (Internet Protocol); this is the type of VPN service that will be used to provide access to general practitioners
- intranet VPNs, which replace private wide area network (WAN) infrastructures, connecting central and remote offices within a corporate intranet with the same policies as a private network; this type of VPN service will be used to interconnect state hospitals, primary health care units, and secondary health care units to the administrative authority of their region
- extranet VPNs, which extend these services beyond the organizations' limits to connect customers and partners, eg, pharmacies and suppliers.

The implementation of the Regional Health Care Data Network's infrastructure should at least initially support intranet and remote-access services based on TCP/IP-protocol-suite standards. This requirement leads to selecting one Internet service provider (ISP) per region, meeting a range of proposed criteria, such as: backbone speed and technology, technical personnel know-how, ability to complete ambitious undertakings, points of presence allocation (number and dispersion) per region and nationwide (that is, extensive network presence), cost-effectiveness, capacity of international links, capability to monitor state-of-the-art VPN solutions, commitment to upgrades and to satisfactory service-level agreements, amount of required protocols and of services offered, efficient management schema, and local customer support.

**Figure 1:**
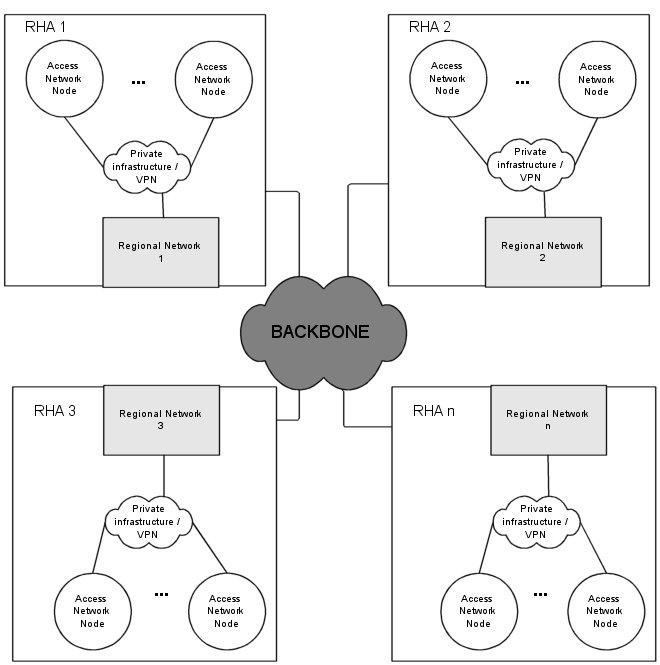
Schematic view of the proposed network infrastructure

We use the term *distribution network* to describe the infrastructure of the ISP selected to implement the VPN solution in a specific region. Distribution network nodes are the points of presence of the ISP in that region. Related health care and administration units should be interconnected and should access the Internet through suitable telecommunication circuits terminating at the ISP's nodes. This will minimize the corresponding installation and maintenance costs of special equipment and specialized personnel. A schematic view of the proposed network infrastructure is shown in Figure 1. Another issue of significant importance is the existence of a suitable access network, ie, a local area network in the health care and administration units' premises, which is presupposed existent and not taken into account within the context of the present study.

Separating the network-infrastructure implementation project into phases should be considered (pilots taking place in technologically-advanced regions would be a reasonable approach). The early stages of such a development timetable are expected to bring out problems associated with the extent that public sector services and relevant contractors are capable of assuming charge of such large VPN solutions.

The commitment of every regional health authority to a single private-sector entity is a key element of the proposed architecture, since selecting more than one ISP would probably create complicated management-related obstacles. The obstacles would originate mainly from compatibility issues emerging when different suppliers' equipment is imposed to ensure end-to-end security, impeding uniform services provision throughout the regional network. The main advantage of the proposed schema utilizing VPN-related technologies is the rapid implementation of the national network infrastructure, focusing on reliability and security, with minimal cost. Given that many regional health care and administrative units are expected to have an active Internet connection by the time the project begins, development will be much easier if some complementary measures are taken, such as selecting one ISP per region and enforcing uniform management procedures (ie, IP addressing and domain name scheme [8]).

Selecting a single entity per region for carrier and service provision gives a broad variety of connection alternatives - Ethernet in the local loop, xDSL (Digital Subscriber Line protocols), ISDN, different types of leased lines - for telecommuters and health care units, depending on the number of active users in each regional health authority. This can also be considered as another step towards reducing telecommunication costs, by enabling the inclusion of regional requirements and characteristics in the overall solution. Even more, the responsibility of maintaining network availability, suitable computer rooms, and guaranteed technical support is shifted to the ISP's specialized personnel through outsourcing. This is a great advantage of the proposed solution, since the Hellenic health care sector lacks specialized information technology personnel [9].

Advanced data communication services, including Voice over IP (VoIP) - technology providing voice-telephony services over IP connections - and telemedicine applications are expected to considerably reduce the functional costs of the Hellenic Ministry of Health and Welfare and improve the quality of services provided. Desirable quality of service, protection of sensitive traffic or data, infrastructure availability, network administration, development of technologies, and advanced data communication services should be retained in a predefined level through commitment to applicable service-level agreements. The requirements included in the corresponding contracts should be stated by the Hellenic Ministry of Health and Welfare in the form of a global service-level agreement, in order for every regional health authority to be in a position not only to refine and adjust it, but also to maintain the appropriate mechanism for ensuring its terms. The constraint of one ISP per region simplifies such monitoring of a service-level agreement.

It must be clear from what was previously discussed that, because of the possibility of different VPNs being implemented by more than one ISP, there should be a central point per region (called a Regional Data Center) to support nationwide information interchange. Suitable configuration of the different ISPs' equipment and the use of common protocols should ensure end-to-end security. The interconnection between regional data centers and the Hellenic Ministry of Health and Welfare Central Service may be dealt with by using private lines or by building another VPN between the Hellenic Ministry of Health and Welfare Central service and the Regional Data Centers, with the alternative of using an existing backbone network in both cases. A possible network is the SYZEFXIS network, a project of the Hellenic Ministry of the Interior, Public Administration and Decentralization, aimed at developing a uniform telecommunications infrastructure and providing the gate to the trans-European network TESTA for the Hellenic Public Administration [10]. Consequently, the functional integration of regional VPNs into a national network should either be done through developing a private high-speed backbone network for the Hellenic Ministry of Health and Welfare or by using VPN technologies. The proper exploitation of such a backbone network is critical for successful overall infrastructure operation, requiring effective technical support and constant monitoring, as well as improvement of the offered networking services to meet the constantly-increasing demands.

**Figure 2:**
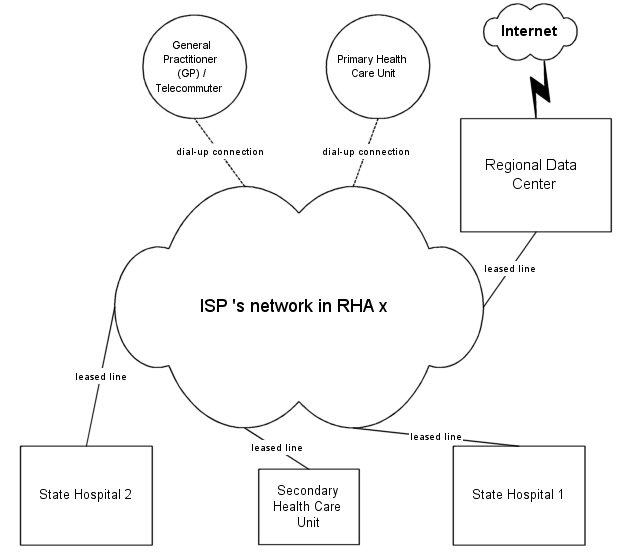
Regional health care data network

Moreover, regional data centers should act as: (1) application service providers (ASPs) hosting necessary applications or data and (2) as concentrators (points where the data streams from many simultaneously active inputs can be combined into one shared channel in such a way that the streams can be separated after transmission) between primary and secondary health care units of their regions and the Internet, with the use of encrypted tunnels (a way to implement a secure link). An obvious advantage of this architecture is the need to develop increased security-protection systems only for this central point of Internet access per region. The corresponding schematic view is depicted in Figure 2.

## Results

Based on the issues previously discussed, the successful development of a secure cost-effective nationwide-network infrastructure for the Hellenic National Health System should be achieved through selecting a proper technology for implementing each regional health authority's VPN and, in a second phase, their functional interconnection. Proper technologies include IPSec (a protocol that provides security for transmission of sensitive information over unprotected networks, such as the Internet, acting at the network layer, protecting and authenticating IP packets between participating devices) or MPLS (a packet-switching protocol). This should be combined with the gradual application of PKI (Public Key Infrastructure) - a system of public key encryption using digital certificates - or other solutions, in the form of pilot projects, ensuring strong authentication, integrity, validity, and non-repudiation.

The ongoing evolution in the way health care is delivered in Greece and corresponding conclusions from several relevant European and international investigations [11- 13] lead us to conclude that the problem of secure interconnection of health authorities should be addressed through regional health care data networks. Therefore each region (or in some cases regions with geographic proximity) should deploy its own data network. The nationwide interconnection should be mainly considered as a functional interconnection, comprising specific services and security characteristics, and not necessarily as a physical linkage between the regional health care data networks and the central authority.

By running the project per region, we achieve independence (eg, a possible failure in a region does not necessarily imply direct effects to others) and life-cycle autonomy, without obstacles originating from time lags due to incompatibilities in penetration of information and communication technologies or from private-sector inability to provide such a solution in a region.

## Discussion

Recognizing the current situation of the Hellenic health care sector, which sums up to:

- low penetration of information and communication technologies
- major reformation in progress,

a study was conducted for the functional interconnection of health care units, to securely exchange medical as well as administrative information. The main characteristics of this study are:

- implementation of regional IP VPNs, based on global service-level agreements with ISPs, including requirements recommended by the Hellenic Ministry of Health and Welfare
- overall integration in a second phase, either through utilizing an existing high-speed backbone network or by using VPN technologies
- a central point per region should support nationwide information interchange to enhance security
- distribution and access networks should be implemented simultaneously.

Since the big challenge is to connect the health care and administration units using public Internet technologies and infrastructures in a secure way, an issue that needs further study is the application of PKI technologies (note that PKI has not been deployed yet on a broad scale in such a complex environment in Europe) [14].

Certain factors should be considered for the Hellenic National Health System to successfully incorporate and exploit the infrastructure proposed in this paper:

- smooth operation; effective administration and expansion
- familiarization of users with the provided network services
- development of new and advanced network services
- improvement of technical know-how and state-of-the-art technology follow-up.

The issues mentioned above, the well-known problem of understaffing of Greek hospitals' Management Information System departments, and the considerable experience gained during relevant former projects, support the idea of engaging outsourcing mechanisms as a viable solution for a reliable and prompt beginning of the infrastructure's productive use.
